# “From chemo to chemo”—the temporal paradox of chemotherapy

**DOI:** 10.1007/s00520-021-06039-6

**Published:** 2021-02-22

**Authors:** Marcin Moskalewicz, Yanna Popova, Jadwiga Wiertlewska-Bielarz

**Affiliations:** 1 Philosophy and Mental Health Unit, Department of Social Sciences and the Humanities, Poznan University of Medical Sciences, Poznan, Poland; 2 Oxford Brookes University, Oxford, UK; 3 Polish Institute of Advanced Studies, Warsaw, Poland

**Keywords:** Phenomenology, Temporality, Lived time, Synchrony

## Abstract

**Purpose:**

To uncover the experience of time in women undergoing chemotherapy for ovarian cancer.

**Methods:**

A combination of consensual qualitative research and Giorgi’s descriptive phenomenology.

**Results:**

The key phenomenon found and pre-reflectively organizing the patients’ experience was the temporal paradox of chemotherapy—a sense of both acceleration and deceleration in between chemotherapy sessions that desynchronizes patients with the time of others.

**Conclusion:**

The experienced paradoxes concentrating around the timings of the chemotherapy treatments are of particular relevance for supportive care. It is particularly important to acknowledge the disturbing effect of the cyclical nature of chemotherapy.

## Introduction

This paper presents a key finding of a study aimed at uncovering the structure of temporal experience in women who undergo chemotherapy for ovarian cancer. Time is a topic that has been notoriously difficult to study. It has also provoked strong disagreements, depending on whether it has been approached by philosophers, physicists or psychologists. This study takes a primarily phenomenological stance, which means that time is understood as human or lived time, not as measured clock time [1]. It allows concentration on how time is directly encountered and experienced by the patients, which is a crucial dimension of their wellbeing. Research on lived time in cancer has been situated within the larger discussion of palliative care [2, 3] or patient experience with advanced-stage cancer [4, 5]. Depending on the age of the patient and stage of the cancer, the studied experience of time includes also various coping strategies for cancer survivors [6, 7]. Some common themes are the experience of hope in patients who have survived [6], active “bracketing away” of negative future while undergoing experimental drug trials [4], and the benefits of maintaining work despite the difficulties of undergoing treatment [7]. Nearly all research has revealed an altered perception of time, manifested most commonly in its changing pace [8], a more explicit temporal awareness [6], living in the moment [4], and in a world with no future [2–8]. When it comes to ovarian cancer, the only temporal theme that emerges from the available qualitative studies is the realization of the aggressive nature of the disease (harsh prognosis, high possibility of recurrence) and the associated likelihood of premature death [9–12].

## Methods

The participants were recruited at a gynaecological oncology ward at a university hospital in Poznan, Poland. Inclusion criteria included a confirmed diagnosis of ovarian cancer and undergoing chemotherapy for at least 6 months. The group consisted of 9 Polish-speaking females between 33 and 52 years of age (mean age 41.7).

The study combined Giorgi’s descriptive phenomenological method with consensual qualitative approach. In Giorgi’s method, first-person qualitative descriptions are broken into units (particular utterances), and then interpretatively transformed into meaning units. Finally, free imaginative variation leads to uncovering what Giorgi calls essential psychological structures, that is general (eidetic) phenomena surpassing first-person descriptions [13, 14]. Semi-structured interviews were taken face-to-face during or just right after a chemotherapy session in between March and May 2019. They lasted between 45 and 90 min each, and were audio-recorded and then fully transcribed. The principle of indirect questioning was applied in order to attend to the participants’ natural attitude (pre-reflective experience) as much as possible. The interviews were coded following the principles of consensual research and subsequently interpreted.

## Results

Three of the ten distinguished meaning units (themes) reveal the temporal paradox of chemotherapy. These themes are as follows: (1) the paradox of time, (2) losing and gaining control over time (both present in all participants), and (3) chemo-clock (present in all but one participant). Altogether, these themes form a pattern that, we may speculate, represents a crucial structural aspect of lived time during ovarian cancer chemotherapy (see Table 1).

**Table 1:** The themes comprising the temporal paradox of chemotherapy

Theme	Meaning	Example
Paradox of time	The confusion stemming from the sense of time both slowing down in the days right after therapy and slipping away later on.	P3: I want to get over it… but not (…) Well, it’s actually the other way round, during illness there is not enough time for everything, wasn’t like that before.
Losing and gaining control over time	The patients’ struggle to find activities that would make time pass more quickly. It is associated with their physical weakness right after chemotherapy session and subsequent recovery, which operates in cycles.	P6: When one feels better, one wants to make up for everything, wants to use the time.
Chemo-clock	The substitution of usual calendar measures of ongoing time, such as days and weeks, with a three-week scheme. This scheme functions as a basic conceptual tool of orientation in clock time.	P4: I have the feeling sometimes that my life goes in three weeks rounds.

The term chemo-clock was chosen to indicate that chemotherapy sessions function in the manner of calendar time conceptual units, which recur in a rhythmic manner. This has to do with the significance of the hospital visits due to which the patients’ life goes in 3-week rounds. (P7: *I have chemo appointments every three weeks, I would like not to think of what will happen (…) Unfortunately one thinks of what will happen, cause this is this disease (…) I live from appointment to appointment*). Another participant asked about whether she locates her plans in calendar time says P8: *No. Unless it’s the next chemo, yes, I have an appointment for the next chemo, then I know it’s three weeks ahead on a such and such day, but otherwise no*. At the same time, in between treatments, one’s life goes up and down. The origin of this presumably lies in the overwhelming physical weakness experienced right after chemotherapy, of which all participants have spoken. One feels that while one’s time drags, the world’s time is running away, and one is trying to regain control over it through various activities. As a result, there is a sense of both acceleration and deceleration, and one is caught in felt temporal contradictions. This represents a variation of the classic retrospective paradox of time, in which time that drags while lived shrinks retrospectively, and time that flies while lived stretches. Here, the paradox of acceleration and deceleration is ongoing, and in this sense prospective.

## Discussion

It is almost surprising how biological rhythms (such as circadian) and conventional conceptual schemes of the clock (hours, days, weeks, months) lose their relevance for the patients. In addition, because of the rhythm and physical impact of chemotherapy, patients become desynchronized with social temporality. They are losing and gaining control over time, the former being an unpleasant, and the latter a pleasant experience. But they are not simply wanting to slow-down the accelerated passage of time, as this passage is both accelerating and slowing down, depending on the stage of the cycle of chemotherapy. Consequently, they are both willing to slow-down and speed-up their lived time in order to strike a balance during their temporal ups and downs in between of chemotherapy sessions. Finally, the loss of social synchrony is accompanied by the establishment of a new synchrony and a new measure of time, termed here the chemo-clock. This chemo-clock temporarily organizes the patients’ life top-down, so to speak, and is the single, most pervasive phenomenon of their temporal experience.

## Conclusion

To our knowledge, the effects of chemotherapy on temporal experience have not received any critical attention, and the results of this study present a viable hypothesis for further research. They also show why lived time should be acknowledged and taken into account by care and nursing practitioners. This is relevant both “subjectively”—when attending to the needs of the patients, and “objectively”—when organizing care and planning appointments and activities in time. The rhythm associated with chemotherapy appears crucial in this respect. It is critical to recognize the loss of synchrony experienced by the patients, and their struggle to strike a balance between felt acceleration and deceleration, and restore them to the present.

## Data Availability

N/A

## Code availability

N/A
